# Robot Fully Assisted Upper-Limb Functional Movements Against Gravity to Drive Recovery in Chronic Stroke: A Pilot Study

**DOI:** 10.3389/fneur.2021.782094

**Published:** 2022-03-08

**Authors:** Marco Caimmi, Chiara Giovanzana, Giulio Gasperini, Franco Molteni, Lorenzo Molinari Tosatti

**Affiliations:** ^1^Institute of Intelligent Industrial Technologies and Systems for Advanced Manufacturing, National Research Council of Italy, Milan, Italy; ^2^Villa Beretta Rehabilitation Centre, Valduce Hospital, Costa Masnaga, Italy

**Keywords:** stroke, upper extremity, rehabilitation, recovery of function, robotics, passive motion, reaching, task oriented training

## Abstract

**Background:**

Stroke is becoming more and more a disease of chronically disabled patients, and new approaches are needed for better outcomes. An intervention based on robot fully assisted upper-limb functional movements is presented.

**Objectives:**

To test the immediate and sustained effects of the intervention in reducing impairment in chronic stroke and to preliminarily verify the effects on activity.

**Methodology:**

Nineteen patients with mild-to-severe impairment underwent 12 40-min rehabilitation sessions, 3 per week, of robot-assisted reaching and hand-to-mouth movements. The primary outcome measure was the Fugl-Meyer Assessment (FMA) at T1, immediately after treatment (*n* = 19), and at T2, at a 6-month follow-up (*n* = 10). A subgroup of 11 patients was also administered the Wolf Motor Function Test Time (WMFT TIME) and Functional Ability Scale (WMFT FAS) and Motor Activity Log (MAL) Amount Of Use (AOU), and Quality Of Movement (QOM).

**Results:**

All patients were compliant with the treatment. There was improvement on the FMA with a mean difference with respect to the baseline of 6.2 points at T1, after intervention (*n* = 19, 95% CI = 4.6–7.8, *p* < 0.0002), and 5.9 points at T2 (*n* = 10, 95% CI = 3.6–8.2, *p* < 0.005). Significant improvements were found at T1 on the WMFT FAS (*n* = 11, +0.3/5 points, 95% CI = 0.2–0.4, *p* < 0.004), on the MAL AOU (*n* = 11, +0.18/5, 95% CI = 0.07–0.29, *p* < 0.02), and the MAL QOM (*n* = 11, +0.14/5, 95% CI = 0.08–0.20, *p* < 0.02).

**Conclusions:**

Motor benefits were observed immediately after intervention and at a 6-month follow-up. Reduced impairment would appear to translate to increased activity. Although preliminary, the results are encouraging and lay the foundation for future studies to confirm the findings and define the optimal dose-response curve.

**Clinical Trial Registration:**

www.ClinicalTrials.gov, identifier: NCT03208634.

## Introduction

The global incidence of stroke is increasing, while at the same time the incidence of death from stroke is declining (1). This means that stroke is transitioning even more into a disease of chronically disabled survivor (2). Each year, 17 million people worldwide suffer from a stroke, and approximately one-third of them present upper-limb impairment still in the chronic stage (3). Robot-assisted training is a relatively novel approach, which in patients with stroke can improve arm and hand function, arm and hand muscle strength, and ultimately their activities of daily living, but the quality of the evidence is still poor (4). Few studies evaluated robotic upper-limb rehabilitation in chronic stroke. The first study on a group of 42 patients with moderate-to-severe chronic impairment demonstrated improved motor abilities after treatment, which were sustained at a 4-month follow-up (5). A more recent study including 20 patients with severe-to-moderate impairment seems to confirm improved function is maintained at a 3-month follow-up (6). However, the added effect of robotic interventions with respect to other therapies is not demonstrated yet. Lo et al. who studied the effect of robotic therapy on 49 patients with long-term severe-to-moderate impairment, did not find significantly improved motor function at 12 weeks, as compared with usual care or intensive therapy (7). They found better outcomes over 36 weeks only compared with usual care and not with intensive therapy. A further subgroups analysis showed that improvements were not homogeneous over the group; younger age and a shorter time since stroke were associated with more significant immediate and long-term improvement of motor function (8). A recent multicentric study on 257 patients with subacute and chronic stroke demonstrated that an intervention based on the planar Massachusetts Institute of Technology (MIT)-robotic arm is not superior to an upper-limb therapy (EULT) program based on repetitive functional-task practice and to usual care (9).

New studies on patients with chronic stroke are needed to get insight into the mechanisms leading to improved motor function following robotic treatment to predict the outcome and define criteria for patients' selection and personalization of therapies. The type and intensity of the robotic intervention and the duration of the rehabilitation program, along with the patient's age, distance from the stroke, and level of impairment, are some of the factors influencing the outcome and are worthy of being studied. Two preliminary studies suggest that interventions with spatial robotic devices, which are able to assist in reaching against gravity could have an additive effect on motor recovery in patients with chronic stroke with moderate-to-severe hemiparesis (10, 11). The authors of the present study proposed a novel robotic approach based on fully assisted functional movements against gravity performed at quasi-physiological velocity. The movements are in the *real world* and cover the entire peripersonal space, moving toward and away from the body. Importantly, they are everyday gestures involving brain emotional processes, like in the case of the hand-to-mouth movement, which may recall eating. The resulting exercises are, therefore, highly engaging and stimulating. The authors found short-term-improved function in a very preliminary study on a group of 10 patients with chronic stroke with mild-to-severe impairment (12). The study reported here expands on the previous research by examining the effects of robotic rehabilitation in a larger sample of subjects with chronic stroke and by investigating on a smaller group of patients whether improved function translates to increased activity in the short term. Further, a preliminary investigation is performed to verify whether improvements in motor abilities are sustained at a 6-month follow-up.

## Methods

### Study Population and Design

In this cohort study, a convenience sample of 19 patients with mild-to-severe upper-limb impairment, 6 months or more post-stroke, were enrolled. The inclusion criteria were: (i) hemiplegia after the first stroke; (ii) time from the stroke event >6 months; (iii) absence of severe attentive deficits; (iv) ability to perform active arm movements (shoulder flexion Medical Research Council [MRC] >1 and Active Range of Movement (AROM) >60°, elbow flexion-extension MRC >1 and AROM >90°) and able to hold the robot handle, and (vi) Modified Ashworth Scale (MAS) score ≤ 3 (refer to section outcome). Exclusion criteria were: (i) other concurrent upper-limb rehabilitation interventions; (ii) presence of global aphasia and/or cognitive impairments that could interfere with understanding the instructions during evaluation and treatment (Mini-Mental State Examination Test>24/30); and (iii) concomitant progressive central nervous system disorders, peripheral nervous system disorders, or myopathies.

**Figure 1:**
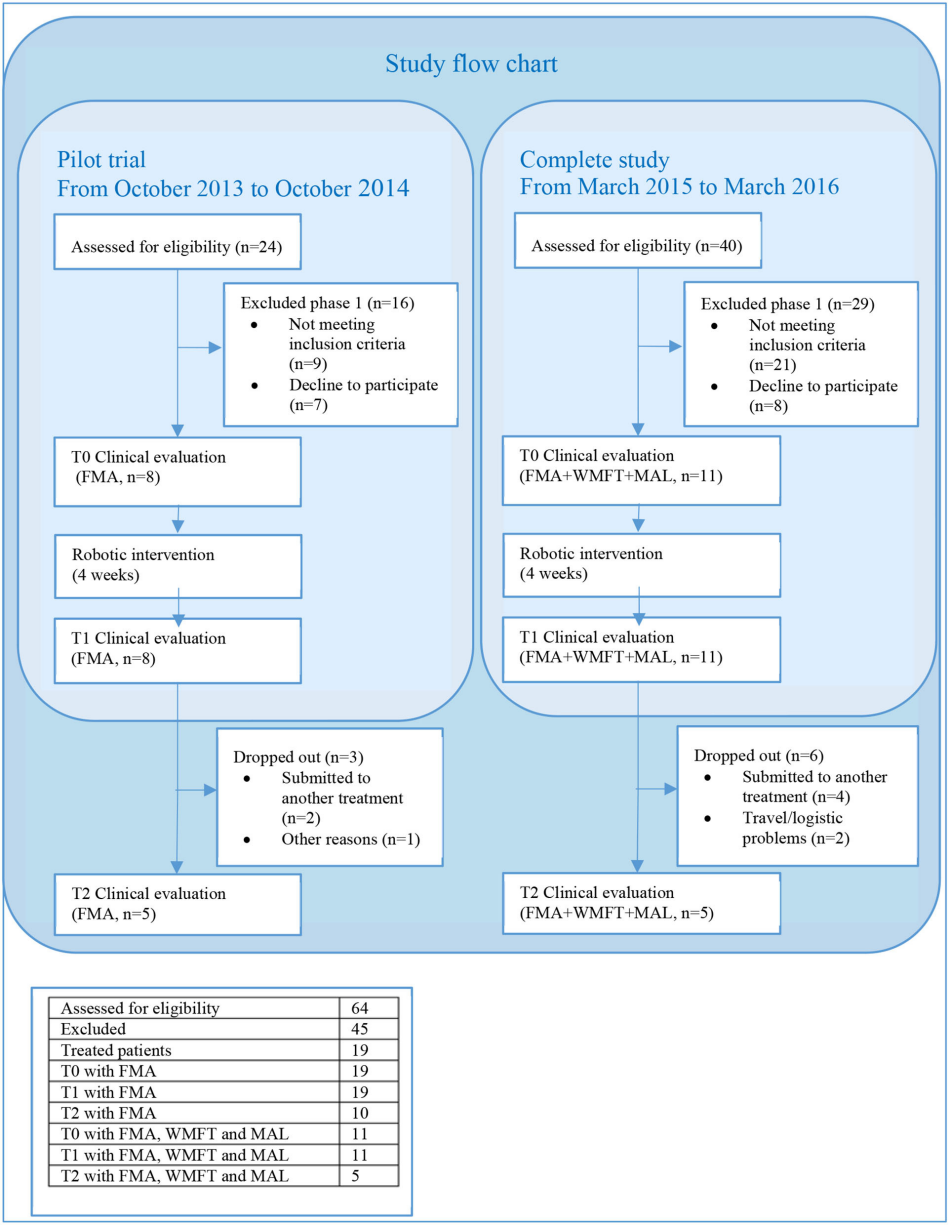
Study flow chart. The pilot trial took place from October 2013 to October 2014. Twenty-four patients selected from the Villa Beretta database were called over the phone and invited to participate in the study. Seventeen agreed and were screened; 5 were excluded, mainly because they were not able to hold the robot handle during one of the two movements, and 7 refused to participate. From March 2015 to March 2016, a total of 40 patients with chronic stroke who were referred to the outpatient clinic of Villa Beretta were screened; 23 were excluded because of not meeting the inclusion criteria (insufficient shoulder and elbow active ROM or inability to hold the robot handle), and 6 refused to participate. The most common reason for refusing to participate referred to difficulties in reaching the facility.

The study was performed in two phases. A pilot trial involving eight patients aimed to verify the short-term efficacy of the robotic intervention in reducing motor impairment. Given the first positive results, the study was completed by recruiting 11 other patients to verify whether functional improvements translate to increased activity. Figure 1 reports the flow chart of the study and a table summarizing the total number of patients who were assessed for eligibility, along with the number of patients who were excluded from the study and the ones who were treated. The local ethics committee granted the study, and all recruited participants provided written informed consent (CE 126 /2011 on 23/09/2011, and amendment CE 219/2014 on 09/10/2014). The study is registered with ClinicalTrials.gov as “Rehabilitation Multi Sensory Room for Robot Assisted Functional Movements in Upper-limb Rehabilitation in Chronic Stroke (RehaMSR),” study ID NCT03208634.

A more comprehensive summary of the study, including a detailed description of the type of assisted movements and training parameters, is presented elsewhere (12); a summary is presented below.

### Intervention

**Figure 2:**
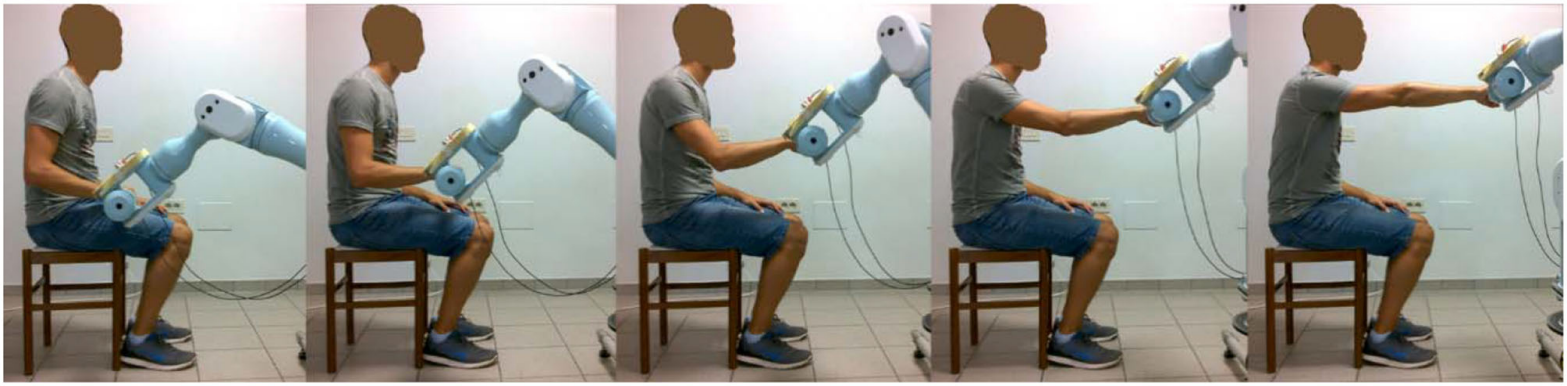
Assisted RM: starting with the robot handle just above the thigh, the assisted Reaching Movement (RM) consisted of compound movements of shoulder flexion and elbow extension, getting as far as 90 degrees of shoulder flexion and fully extended elbow were reached.

**Figure 3:**
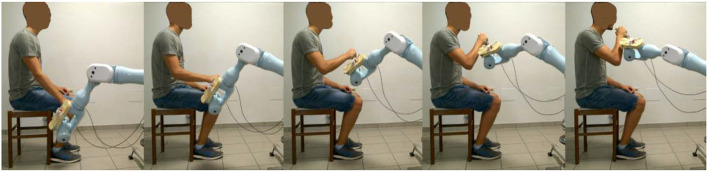
Assisted Hand-to-Mouth Movement (HtMM): Starting with the robot handle just above the thigh, the assisted HtMM consisted in flexing the elbow (and the shoulder) to position the robot-handle in front of the mouth. Importantly, the handle was free to rotate and, therefore, the patient had to put it actively (performing wrist internal/external rotation movements) in the right position, which was with its extremity pointing toward the mouth.

The intervention was administered by a trained research therapist *via* an end-effector robot (Pa10-7, Mitsubishi, Japan), which was customized to assist 3D multi-joint functional movements against gravity performed at physiological velocity. The intervention protocol, identical in the two phases of the study, consisted in the execution of two functional movements, namely the Reaching Movement (RM) against gravity (Figure 2) and the Hand-to-Mouth Movement (HtMM, Figure 3). Each session consisted of 20 min of robot-assisted RM and 20 min of robot-assisted HtMM. The movements were fully assisted (the robot handle moved along the path with a predefined motion law independently of the forces exerted on the handle), but the patient was explicitly asked to participate by trying to follow (slightly anticipate) the moving handle. During movement, the operator could check on a monitor the forces applied on the robot handle and encourage the patient to participate more in the movement. The movement is fully assisted because, in essence, it is performed following the preselected motion law even in the case of a plegic arm; however, the patient is free/is asked to try to perform the movement on his own. When the patient is (partially) able to perform the movement, the robot has the function to drive the patient in following the right path and perform the movement smoothly at the preselected pace.

The rehabilitation consisted of a 1-month intervention, three sessions a week performed on Monday, Wednesday, and Friday, for 12 sessions in total.

### Clinical Assessment

The patients selected for the study were clinically tested at baseline (T0), just after intervention (T1), and at a 6-month follow-up (T2). The patients who underwent other interventions between T0 and T1 or T1 and T2 were excluded from the evaluation at T1 or T2, respectively. One trained physical therapist, the same for all patients, performed all outcome assessments (pretreatment as well as post-treatment and follow-up) with the supervision of the patient's referent physician. To minimize biases, the patient could not have access to and view the results of the previous sessions.

The primary outcome measure was the upper-limb motor function subdomain of the Fugl-Meyer Assessment (FMA) (14) made of the sections A-D (A shoulder and elbow, B wrist, C hand, and D coordination of the upper limb). The secondary outcome measures were the Wolf Motor Function Test Time (WMFT TIME) (15) and Functional Ability Scale (WMFT FAS) (16), and the Motor Activity Log (17) Quality of Movement (MAL QOM) and Amount Of Use (MAL AOU), which were administered to 11 out of the 19 patients, that is only in the final trial. The MRC scale for muscle strength (18) and the MAS (19) were administered to complete the patients' clinical picture. Finally, a qualitative analysis of the Draw-A-Person test (20) was performed to assess the possible deficits of the mental body representation (21).

### Data Analysis

Based on the primary outcome measure results of the pilot trial, the sample size of the study was computed using the G^*^Power 3.1.9.2 statistical power analysis program (22). The results referred to a sample of 19 patients would allow detecting a seven-point FMA improvement with an SD of 11.3 (medium effect size, Cohen *d'* >0.60), 80% power, and a type I error of 0.05.

For analysis, the patients were clustered in groups based on the number of evaluation sessions and the type of clinical assessments they underwent. Comparisons of the same groups' data between different evaluation sessions were performed with the Wilcoxon signed-rank test, considering the value of significance at 0.05. Because of the small size of the sample, a non-parametric test was preferred. The responsiveness of the clinical measures to the intervention was estimated using Cohen's effect size (d') (23, 24). Linear regression and Pearson's correlation and Evans' classification (25) were used for verifying and quantifying possible correlations between the FMA improvements at T1 and the age of patients, the time from the stroke, and the level of impairment at T0. For this analysis, improvements were calculated both as absolute improvements, i.e., ΔFMA = FMA_T1_ -FMA_T0_, and improvements normalized to the maximum potential recovery (26): ΔFMA_NOR_ = ΔFMA/(66-FMA_T0_). The statistical analysis was performed using WinSTAT® ver.2012.1.0.94.

## Results

Nineteen patients participated in the study, 11 underwent additional WMFT and MAL, and 10 reached the evaluation at T2; their data are reported in Table 1. Only five patients of the group with additional WMFT and MAL reached T2 (refer to Figure 1); the results are not reported as an apart group in Table 1 as the sample is too small to draw any conclusion. The mean age of the participants was 62 years (±2^*^SE = 9 years), and the time from stroke was 45 months (±2^*^SE = 30 months); six (32%) were women, 11 (58%) had right hemiparesis, seven (37%) had a hemorrhagic stroke, and all other patients had an ischemic one. At baseline, the patients had mild-to-severe impairment according to the classification of Woytowicz (27): Three were severely impaired (FMA <29), six were moderately impaired (29 ≤ FMA ≤ 42), and 10 were mildly impaired (FMA >42). The Draw-A-Person test showed heterogeneous results at T0; two thirds of the patients' drawings showed at least an anomaly; the most common anomalies were a lack or disproportion of body segments (hands, feet, and entire upper and lower limbs) and a lack of facial elements (mouth, nose, eyes, and ears). All patients were fully compliant with the treatment and could complete all 12 robotic rehabilitation sessions. In average, the patients performed 338 (218-400) RM and 238 (170-340) HtMM each session. The average number of reaching and hand-to-mouth movements performed by patients after the 1-month treatment was 6918 (6420–8160). No adverse effect was observed; the intervention is safe in this sense. Unfortunately, nine out of the 19 patients who underwent the treatment missed the clinical evaluation at T2, mainly because they received a medical treatment between T1 and T2, which could interfere with the clinical evaluation results. The clinical results at T0, T1, and T2, along with the Cohen's effect size d' and *p*-values, are reported in Table 2.

**Table 1:** Patients' data.

**Patients**	**Age (years)**	**Sex**	**Affected side**	**Stroke type**	**Stroke location**	**Time from stroke (months)**	**Nr of RM**	**Nr of HtMM**	**Nr total movements**
Pt 1	65	F	Left	Ischemic	Right lenticular nucleus and internal capsule	6	360	245	7,260
Pt 2	62	M	Right	Hemorrhagic	Left caudate nucleus and internal capsule	76	340	240	6,960
Pt 3	24	F	Right	Hemorrhagic	Left frontal lobe	32	337	210	6,564
Pt 4	65	M	Left	Ischemic	Right Frontoparietal lobe	11	330	230	6,720
Pt 5	76	F	Right	Ischemic	Left hemisphere	27	350	240	7,080
Pt 6	68	F	Right	Hemorrhagic	Left basal ganglia	51	330	210	6,480
Pt 7	55	M	Left	Ischemic	Right temporal lobe	32	315	245	6,720
Pt 8	65	M	Right	Ischemic	Left hemisphere	6	400	260	7,920
Pt 9	73	M	Left	Ischemic	Right basal ganglia	8	310	250	6,720
Pt 10	49	M	Right	Ischemic	Left frontoparietal lobe	19	310	212	6,264
Pt 11	74	M	Left	Hemorrhagic	Right semioval center and left frontobasal lobe	10	320	220	6,480
Pt 12	67	M	Right	Ischemic	Left thalamus	6	350	230	6,960
Pt 13	66	M	Left	Ischemic	Right hemisphere	66	218	340	6,696
Pt 14	46	F	Right	Ischemic	Left parahippocampal gyrus	168	345	205	6,600
Pt 15	64	M	Left	Hemorrhagic	Right basal ganglia	112	365	170	6,420
Pt 16	56	M	Right	Hemorrhagic	Left frontoparietal lobe	151	360	255	7,380
Pt 17	35	F	Right	Ischemic	Left frontoparietal lobe	44	400	280	8,160
Pt 18	80	M	Left	Ischemic	Right posterior internal capsule	27	340	230	6,840
Pt 19	82	M	Right	Hemorrhagic	Left Internal capsule	8	348	241	7,068
	62 ± 9	6 F	11 Right	12 Ischemic	-	45 ± 30	338 ± 24	238 ± 21	6,910 ± 304

*Nr of RM, average number of assisted Reaching Movements performed at each training session; Nr of HtMM, average number of assisted Hand to Mouth Movements performed at each training session; Nr total movements, RM + HtMM performed in total during the 12 training session*.

**Table 2:** Means ± Double SEs for clinical results along with T1 vsT0 and T2 vsT1 Cohen's Effect Size d' and *p*-values.

**Evaluation**	**T0**	**T1**	**T2**	**Immediately after intervention**			**Six months after intervention**		
				**Mean diff (95% CI)**	**d'**	***p***	**Mean diff (95% CI)**	**d'**	***p***
**(*****n*** **= 19)**									
FMA	42.8 ± 5.7	48.9 ± 5.1		6.2 (4.6–7.8)	0.55	<0.0002			
FMA SecA	25.2 ± 2.6	28.3 ± 2.4		3.1 (2.2–4.0)	0.60	<0.0005			
FMA SecB	5.3 ± 1.4	5.9 ± 1.5		0.7	0.21	ns			
FMA SecC	8.1 ± 1.9	10.1 ± 1.7		2.1 (0.9–3.2)	0.57	<0.001			
FMA SecD	3.8 ± 0.5	4.6 ± 0.5		0.8 (0.6–1.1)	0.86	<0.001			
MRC	10.2 ± 0.7	11.3 ± 0.7		1.1 (0.6–1.5)	0.36	<0.002			
MAS	3.4 ± 0.7	3.1 ± 0.8		−0.3	0.25	ns			
**(*****n*** **= 11)**									
WMFT TIME	7.3 ± 1.5	6.5 ± 1.3		−0.8s	0.38	ns			
WMFT FAS	4.0 ± 0.3	4.3 ± 0.3		0.3 (0.2–0.4)	0.67	<0.004			
MAL AOU	1.46 ± 0.71	1.64 ± 0.74		0.18 (0.07–0.29)	0.15	<0.02			
QOM	1.29 ± 0.71	1.43 ± 0.72		0.14 (0.08–0.20)	0.12	<0.02			
FMA	48.0 ± 5.1	53.9 ± 4.1		5.9 (4.0–7.8)	0.88	<0.004			
FMA SecA	28.1 ± 2.2	31.1 ± 1.6		3.0 (1.9–4.1)	1.13	<0.006			
FMA SecB	5.8 ± 1.6	6.3 ± 1.9		0.5	0.14	ns			
FMA SecC	9.9 ± 1.9	11.5 ± 1.5		1.6 (0.7–2.4)	0.64	<0.02			
FMA SecD	4.2 ± 0.4	5.1 ± 0.4		0.9 (0.5–1.3)	1.36	<0.02			
MRC	11.5 ± 0.5	12.5 ± 0.5		1.0 (0.5–1.3)	0.54	<0.02			
MAS	3.7 ± 1.5	3.7 ± 1.7		0.0	0.00	ns			
**(*****n*** **= 10)**									
FMA	38.4 ± 9.1	44.3 ± 7.2	48.1 ± 7.2	5.9 (3.6–8.2)	0.47	<0.005	9.7 (4.6–14.8)	0.86	<0.007
FMA SecA	23.2 ± 4.3	25.9 ± 3.6	27.8 ± 3.4	2.5 (1.2–3.7)	0.47	<0.02	4.6 (1.5–7.7)	0.86	<0.02
FMA SecB	4.4 ± 2.2	5.1 ± 1.9	5.6 ± 2.1	0.7 (0.2–1.2)	0.23	<0.05	1.2 (0.2–1.2)	0.36	<0.05
FMA SecC	6.5 ± 3.0	9.0 ± 2.8	10.3 ± 2.6	2.5 (0.6–4.4)	0.57	<0.02	3.8 (1.5–6.1)	0.92	<0.008
FMA SecD	3.3 ± 0.8	4.3 ± 0.8	4.4 ± 0.7	1.0 (0.6–1.4)	0.84	<0.02	1.1 (0.5–1.7)	0.99	<0.02
MRC	8.9 ± 1.0	9.9 ± 1.1	11.8 ± 0.7	1.0 (0.5–1.3)	0.28	<0.02	2.9 (0.6–3.9)	1.07	<0.006
MAS	3.4 ± 0.8	2.8 ± 1.1	2.6 ± 1.4	−0.6	0.47	ns	−0.2	0.09	ns

*The table is in 3 parts: (1) FMA of 19 patients at T0 and T1; (2) WMFT and MAL at T0 and T1 of a subgroup of 11 patients (along with FMA to define the level of impairment) and (3) FMA at T0, T1 and T2 of a subgroup of 10 patients. MRC and MAS are shown to complete the patients' clinical picture. FMA, Upper-Extremity Fugl-Meyer Assessment (max 66 pts); SecA, Shoulder and Elbow Section (max 36 pts); SecB, Wrist Section (max 10 points); SecC, Hand Section (max 14 pts); SecD, Coordination/Velocity Section (max 6 pts); MRC, Medical research Council (max 15 points); MAS, Modified Ashworth Scale (max 15-negative points); WMFT, Wolf Motor Function Test; TIME, average duration (s) to perform items; FAS, Functional Ability Scale (max 5 pts): MAL, Motor Activity Log; AOU, Amount of Use (max 5 points); QOU, Quality Of Use (max 5 pts)*.

**Figure 4:**
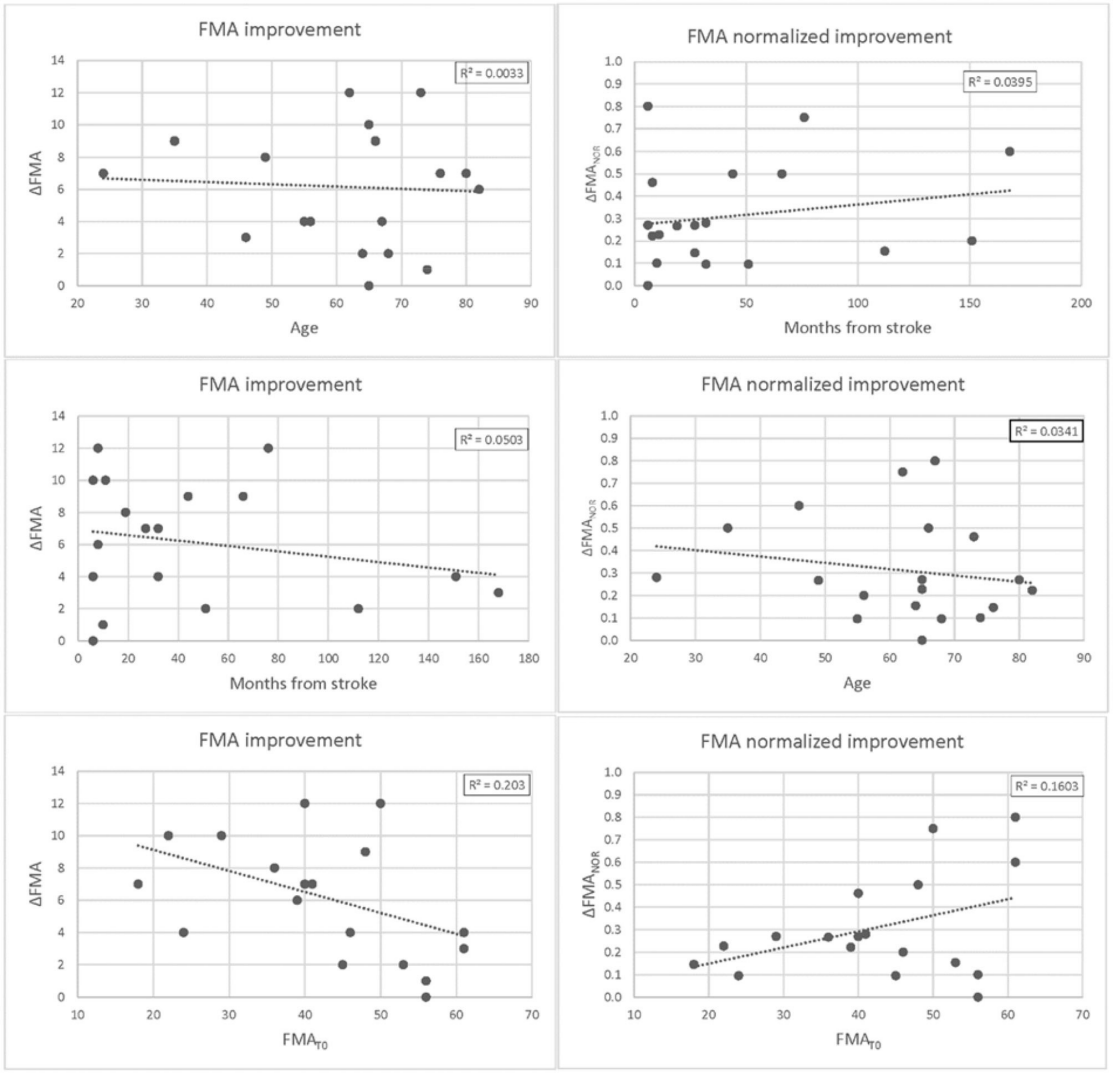
Differences in Fugl-Meyer Assessment (FMA) at T1 vs. T0 plotted against patients' age (**upper** panel), months from stroke (**middle** panel), and FMA scores at baseline. Differences are expressed as absolute values ΔFMA = FMAT1 -FMAT0 (**left** panel) and potential recovery ΔFMANOR = ΔFMA/(66-FMAT0) (**right** panel). For each plot, the linear regression curve along with the r-squared value is also shown.

**Figure 5:**
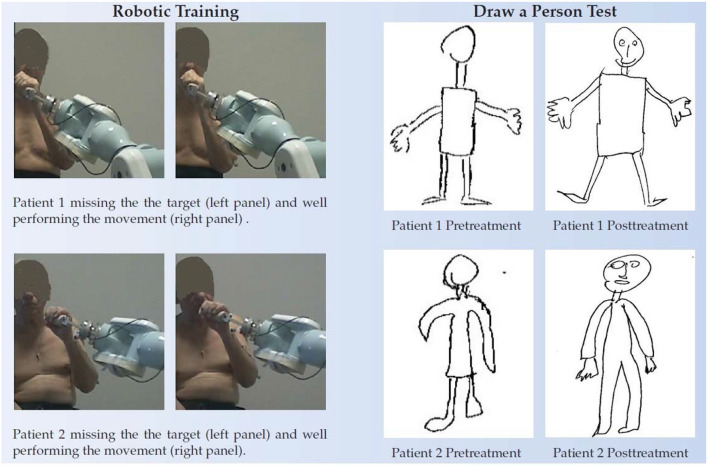
Draw-A-Person test of two chronic patients. **Left** panel, the two patients performing the robot assisted movements (first trials very left pictures) and after some sessions of training. In the beginning, they were not able to place the robot handle in front of the mouth as requested. **Right** panel, the pre and posttreatment Draw-A-Person test.

At T1, the patients (*n* = 19) showed a statistically significant improvement in the FMA (+6.2/66, 95% CI = 4.6–7.8, *p* < 0.0002) as well as almost all the FMA subsections (the shoulder and elbow–SecA +3.1/36, 95% CI = 2.2–4.0, *p* < 0.0005; the hand–SecC +2.1/14, 95% CI = 0.9–3.2, *p* < 0.001; and the coordination–SecD +0.8/6, 95% CI = 0.6–1.1, *p* < 0.001). The improvement (+0.7/10) in section B score, the one regarding the wrist functionality, was not statistically significant (*p* = 0.075). Regression curves are plotted in Figure 4. There was no correlation neither between ΔFMA and the patients' age (r = −0.06, *p* = 0.40) nor between ΔFMA and the time from stroke (r = −0.22, *p* = 0.18). The same results were found when the ΔFMA normalized on the maximum potential recovery was used for regression (ΔFMA_NOR_ *vs*. “age”: r = −0.18, *p* = 0.22; ΔFMA_NOR_ *vs*. “time from stroke”: r = −0.20, *p* = 0.21). A moderate negative correlation was found between ΔFMA and the patients' functional level at baseline FMA_T0_ (r = −0.45, *p* < 0.03) but, by contrast, a moderate positive correlation was found between ΔFMA_NOR_ and FMA_T0_ (r = 0.40, *p* < 0.05). As regards the MRC, there was a statistically significant increase in the score (+1.1/15,95% CI = 0.6–1.5, *p* < 0.002) while the reduction of 0.3 in the MAS score was not statistically significant. Responsiveness to the intervention, according to Cohen's definition, was large for FMA SecD, moderate for FMA, FMA SecA, and FMA SecC, and small for MRC. The qualitative analysis of the drawings of the Draw-A-Person test that presented some anomalies at T0 showed heterogeneous results, ranging from no-difference to the full integration of all body segments and recovery of their proportion at T1 compared with T0. In Figure 5, two self-explanatory examples show the recovery of the facial elements, body segments, and proportions.

At T1, the subgroup of patients (*n* = 11) who underwent additional WMFT and MAL evaluation, showed a statistically significant improvement in WMFT FAS (+0.3/5, 95% CI = 0.2–0.4, *p* < 0.004) but not in WMFT TIME (−0.8s, *p* > 0.05). They showed a statistically significant improvement in the quantity (AOU, +0.18/5, 95% CI = 0.07–0.29, *p* < 0.02) as well as the quality scale (QOM, +0.14/5, 95% CI = 0.08–0.20, *p* < 0.02) of the MAL. Also, for this group, improvements in FMA total score and subsections A, C, and D were statistically significant (FMA +5.9/66, 95% CI = 4.0–7.8, *p* < 0.004; SecA +3.0/36, 95% CI = 1.9–4.1, *p* < 0.006; SecC +1.6/14, 95% CI = 0.7–2.4, *p* < 0.02; and SecD +0.9/6, 95% CI = 0.5–1.3, *p* < 0.02) as well as in MRC (+1.0/15, 95% CI = 0.5–1.3, *p* < 0.02). Responsiveness to the intervention was large for FMA total score and subsections A and D, moderate for WMFT FAS, FMA SecC, and MRC, and trivial for MAL, both quantity and quality of use.

The subgroup of patients (*n* = 10), who reached the evaluation at T2, showed statistically significant improvements at T1, in FMA total score and all subsections and MRC (FMA +5.9/66, 95% CI = 3.6–8.2, *p* < 0.005; SecA +2.5/36, 95% CI = 1.2–3.7, *p* < 0.02; SecB + 0.7/10, 95% CI = 0.2–1.2, *p* < 0.05; SecC + 2.5/14, 95% CI = 0.6–4.4, *p* < 0.02; SecD +1.0/6, 95% CI = 0.6–1.4, *p* < 0.02; MRC + 1.0/15, 95% CI = 0.5–1.3, *p* < 0.02). Comparison between T1 and T2 results showed a statistically significant improvement in MRC (+1.9/15, *p* < 0.02, responsiveness = 0.62) while improvements in FMA total score and subsections were not significant. Comparison between T0 and T2 showed statistically significant improvements in all FMA subsections (FMA +9.7/66, 95% CI=4.6-14.8, *p* < 0.007; SecA +4.6/36,95% CI=1.5-7.7, p < 0.02; SecB +1.2/10, 95% CI=0.2-1.2, *p* < 0.05; SecC +3.8/14, 95% CI = 1.5–6.1, *p* < 0.008; and SecD + 1.1/6, 95% CI = 0.5–1.7, *p* < 0.02) as well as MRC (+2.9/15, 95% CI = 0.6–3.9, *p* < 0,006). The responsiveness to the intervention measured at T1 was large for FMA SecA, moderate for FMA SecD, and small for FMA total score, SecB, and SecC as well as MRC. The responsiveness to the intervention measured at T2 was large for all scales but FMA SecB, which was small.

In the last subgroup (*n* = 5), made of the subjects who underwent additional WMFT and MAL evaluation and reached T2, 2 subjects showed improvements compared with T1, while the other three showed no difference. The result is encouraging, but the sample size is too small to draw any conclusion on the patients' activity 6 months postintervention.

## Discussion

The present study investigated the short-term effects of a novel robotic intervention in a group of patients with chronic stroke by evaluating the impairment reduction measured through FMA and aimed to verify in a subgroup of patients whether improved function translates to increased activity by evaluating the WMFT and the MAL. Further, a preliminary analysis of the FMA was performed in the second subgroup of patients who reached the evaluation at T2 to verify whether improvement reduction is maintained at a 6-month follow-up.

The results showed improved group average overall upper-limb function following the intervention that can be considered both real and clinically meaningful for people with chronic stroke as ΔFMA is >6 points and, therefore, beyond both the Minimal Detectable Change (MDC) and the Minimal Clinically Important Difference (MCID), which for the FMA are both equal to 5.2 points (28).

The intervention is relatively short (4 weeks) and relatively frequent (3 times a week); however, it is intense (577 movements each session, 6,918 in a month, on average). Previous studies on chronic stroke reported do differ for the duration [Fasoli et al. (5) and Posteraro et al. (6): 3 times a week for 6 weeks; Lo et al. (7): 36 1-h sessions over a period of 12 weeks; Rodgers et al. (9): 3 times a week for 12 weeks]. It is further important to recall that these results were obtained with an intervention based on fully assisted movements. Therefore, the approach neither meets the *assist as need* principle nor the *Detection of Patient Intent* (DPI) method, used in robotic rehabilitation to maximize neuroplasticity (12). However, a recent neurophysiological study by the group of Farina demonstrated that Hebbian plasticity could be induced in healthy subjects even by using a passive device, as long as motor imagery is combined (29). Our patients were explicitly asked to (try to) follow the movement of the robot handle they were grabbing during repeated RM and HtMM. So, they knew the starting time of the next movement, and similar to the study of Farina et al. they were imagining the movement just before it started and, conceivably, Hebbian plasticity mechanisms could have been enhanced. This needs further investigations as, on the one side, Farina's results were on healthy subjects and, on the other side, the veracity of the neurophysiological equivalence between his study and ours is not sure. However, there is evidence that Hebbian-type stimulation is feasible even in chronic stroke during robot-assisted wrist movement (30). In addition, and even more importantly, the hypothesis is consistent with a previous study we made in a patient with chronic stroke who was administered the same intervention; we found no organized electroencephalography activity during no-assisted HtMM performed with the affected limb and, conversely, quasi-physiological activity during fully assisted HtMM (31). A specific feature of the hand-to-mouth movement is that the subject is required to orient the handle toward the mouth actively. Therefore, implicitly, the patient is asked to focus on the whole movement and the position of the different body segments. This exercise recalls the *Cognitive Multisensory Rehabilitation*, a promising therapy for upper limb recovery in stroke, where the therapist probes the patients through questions to consciously reflect on the position of their arm and hand in space and to have a focused awareness to the multisensory processing and their movements during sensory discrimination exercises. In a recent study, the authors explained that Cognitive-Multisensory-Rehabilitation exercises target the restoration of body awareness directly, which in turn, directly and indirectly, improves body image and, thus, *Mental Body Representation* as a whole, which aids the restoration of motor function (21). At the brain function level, they found, following the intervention, increased functional connectivity in the parietal operculum between the right OP1/OP4 and 30 areas distributed across all lobes (34 areas were impaired at baseline). In conclusion, they speculated that (i) OP1/OP4 in the multimodal integration network plays a crucial role in the formation of accurate body awareness and that (ii) improvement of body awareness may activate OP1/OP4, leading to restoration of the brain connectivity that was observed. In this study, the patients were administered the Draw-A-Person test to assess mental body representation. The results showed improvements in the posttreatment tests; specifically, the persons drawn were complete with all the body segments, which also respected the proportions; face elements, such as the eyes, the nose, and the mouth, were added with respect to baseline (Figure 5). Even if not investigated, it is conceivable that these patients had decreased connectivity in the brain relevant for sensorimotor function. The intervention could have improved body awareness and, consequently, motor restoration, as described by Van de Winckel et al. (21). However, this hypothesis is not supported by an instrumental evaluation based on functional MRI. It would be interesting to investigate further in a group of selected patients assessing functional connectivity pre and post-intervention.

Interestingly, with the above consideration, improvements were found not only proximally, at the shoulder and the elbow (FMA SecA), but even distally, hand opening included (FMA SecC). First, this was unexpected, considering the robot did not mobilize the hand. The patients had to hold the robot handle during the execution of the assisted functional movements, and this could explain improved finger flexion but not improved hand opening. However, it is known that hand control is affected by the proximal joints position and, particularly, volitional finger extension is, in patients with stroke, affected strongly by shoulder abduction (32). A recent neurophysiological study showed that the development of abnormal joint coupling (flexion synergies) and hand impairment following stroke is correlated with structural changes in the brainstem (33). Possibly, the execution of spatial multi-joint functional movements could help to improve movement velocity, inter-joint coordination, and regaining physiological synergies; in fact, the responsiveness to the treatment was large for FMA SecD (coordination/velocity) and moderate for FMA SecA (Shoulder and elbow). Improved shoulder function and reduction of flexor synergies would explain improved hand opening in our patients. The whole mechanism could also have been enhanced by increased body awareness, as described above. Further investigations based on neuroimaging are needed to confirm these hypotheses.

Different from a previous study by Wu et al. (8), we found no correlation between ΔFMA and the patients' age. There are several possibilities explaining this inconsistency. Simply, our result could be affected by the small size of the sample, or, alternatively, differences in the level of impairment at baseline could account for the result found. In fact, in this study, 10 out of 19 patients presented mild upper-limb impairment; conversely, in the study of Wu et al., the patients had moderate-to-severe upper-limb impairment. A final possible explanation is that motor improvements following our intervention might be due to some biological mechanisms, which are actually independent of the patients' age. This would not be surprising considering that similar results were found post robotic intervention in 190 patients in the subacute phase of the stroke (34); within 6 months, the upper-limb impairment resolves by a fixed proportion of 70% of each patient's maximum possible improvement (35). This “rule,” known as “proportional recovery” holds across all ages, indicating that the motor recovery is due to fundamental biological mechanisms (26). Possibly, even in chronic stroke, other factors like the lesion size and location and involved biological mechanisms play a more critical role in the recovery process than the patients' age. In acute stroke, Byblow et al. demonstrated that 30% of patients do not fit the “proportional recovery rule” because of damage to descending motor pathways (36), and similar results were found in chronic stroke (37).

Furthermore, no correlation was found between ΔFMA and the time from the stroke. This suggests that in the chronic stage of the disease, the mechanisms leading to improved function are active even many years after the stroke. We found 2–4 points of improvement in FMA even 9 years or more after stroke, corresponding to 15–60% of the potential recovery. Finally, all patients except one gained at least 1 FMA point, and 11 patients overtook the MCID. These results are consistent with what Dobkin reported in a review, namely that many patients retain latent sensorimotor function that can be realized any time after stroke with a pulse of goal-directed therapy (38). This is a crucial finding in rehabilitation, also emerging due to the diffusion of robotics that, even more importantly, helps to understand the mechanism leading to the recovery.

The results showed a moderate negative correlation between ΔFMA and the patients' functional level at baseline FMA_T0_; this could be due to the ceiling effect. Indeed, by contrast, a moderate positive correlation was found between ΔFMA_NOR_ and FMA_T0_. The sample size is too low to draw any conclusion regarding a possible correlation between functional recovery and level of impairment at baseline.

About the second question, whether improved function translates to increased activity, the clinical tests seem to confirm the hypothesis. Preliminary results referred to increased activity measured on the WMFT FAS, the responsiveness of which to the treatment was large. However, although the average group improvement (+0.3/5 points) was larger than the MDC, which is 0.1/5 points for patients with chronic stroke, (39) we do not know whether the improvement can be considered clinically meaningful because the MCID is not established yet for patients with chronic stroke. The improvement in WMFT TIME (−0.8 s) was largely beyond the MDC (0.1 s) but was not statistically significant. Similarly, the self-reported arm use has improved as measured on the quantity as well as the quality scale of the MAL, but the responsiveness was trivial for both measures. We can say little about the soundness of the results, as the MDC is not established yet for MAL. It is worth recalling that the intervention was short (1 month, only) and, at T1, the patients could not have realized yet they could perform some daily life activities and, consequently, the validity of the self-reported activity could be affected. Probably, a test at 3- or 6-month follow-up could be more reliable. Unfortunately, out of the 11 patients who were evaluated for WMFT and MAL, only five reached the assessment at T2; the sample is too small to draw any conclusion.

Regarding the last question, whether improvements in motor abilities are sustained at 6-month follow-up, the results are positive. Comparison between the assessments at T1 and T2 showed improvements in FMA total score and all subsections, although they were not statistically significant. However, and even more importantly, improved function in the period T1-T2 was demonstrated by increased responsiveness, which was large for FMA total score and all subsection at T2, whereas at T1, it was moderate for FMA SecD, small for FMA total score, SecB, and SecC, and large only for FMA SecA. Recalling that no intervention was performed in the period T1-T2, it means that no continuous treatment is required, and cyclic treatment sessions can be enough. This is not surprising; probably improvements at T2 were due to increased use of the upper limb in everyday life, as demonstrated by improved MAL and WMFT at T1. This would also explain the moderate improvement in strength as shown by the comparison between the MRC results at T1 and T2 (MRC + 1.0/15, p < 0.02; *d'* = 0.62). To summarize, there is some evidence that the intervention improves both function and upper-limb use in everyday life that further generates a virtuous circle, whereby an increased use of the limb leads to improved function and strength, which, in turn, leads to subsequent increased use and so on. Some preliminary evidence on improved body awareness following the intervention supports this hypothesis. Immediately after the first training sessions, some patients referred the operator to be very satisfied with the intervention because they could move the arm they had not been able to move since the stroke. Although they knew they were performing RM and HtMM not autonomously but, by contrast, with the robot assistance, the movement sensation was so intense as if they were performing the movement themselves. In patients like these, the Draw-A-Person test showed improved body awareness (40). As patients become more aware of their upper limb, they begin to use it more and more in activities of daily living, thus reducing the risk for the *learned non-use* phenomenon.

A final matter to discuss regards the novelty of the approach we used. We already pointed out that the movements were fully assisted and, therefore, there is a risk of negative effects like decreased patient effort and reduced need to “solve” the problem of relearning upper-limb control (11). Conversely, assistance may also have positive effects like increased somatosensory stimulation, complete and accurate proprioceptive signals, better engagement, better ability to do tasks and, thus, receiving positive feedback about efforts. A key goal for robotic therapy device research is to increase the positive effects while decreasing the negative ones (11). In this framework, we believe that movements, highly functional, such as the HtMM may enhance the positive effects and, conversely, reduce the negative ones. The execution of movements that the patient has been repeated many times in life probably recalls intense sensations related to the accomplished task (e.g., eating and/or touching the face) with high emotive impact. In fact, all patients have been able to perform a high number of movement repetitions at each session without ever showing any sign of boredom. In the case of the HtMM, the patients had to position the robot handle in front of the mouth actively. Combining distal active movements (hand tasks) with proximal (elbow and shoulder) fully assisted movements could further be a possible solution to increase the positive effects of robotic assistance and to avoid the negative ones. Even this specific aspect of the intervention that represents a novelty should be a matter of discussion in future studies. To the best of our knowledge, no other studies have been done on a rehabilitation approach based on robot-assisted functional movements in the peripersonal space performed at quasi-physiological velocity and, therefore, with high smoothness. Future studies may verify whether these novelties in the field of rehabilitation robotics could help to maximize the results. Particularly interesting would be to confirm the results that demonstrated increased activity, which actually is the ultimate goal of post-stroke rehabilitation. In fact, at the state of the art, the efficacy of robotic rehabilitation in increasing arm use is still a matter of discussion as no sound evidence has been found yet (4).

### Limitation of The Study

There are several limitations to our study. First, the sample size is small, and there are several dropouts, particularly at the follow-up, as some patients, given the good results, started other rehabilitation programs before being tested at the six-month follow-up. Therefore, the intervention will need to be applied to a larger sample of participants to confirm the results found: significant improvements in the function of the shoulder, elbow, and hand, in movement velocity, and inter-joint coordination as measured on the upper-limb FMA scale. Second, as there was no control group, we cannot know whether patients have improved better than they could have following other therapies (*e.g.*, traditional therapy, constraint-induced movement therapy, or other robotic therapies). Anyway, this study is preliminary, and the aim was to verify the efficacy of the intervention to justify a future randomized control trial. Third, we do not know whether a more intensive and more prolonged treatment could have led to better results. However, in the field of robot-assisted therapy, this is still a general open question to be further investigated (2). Very few trials have looked into the optimum intensity and duration of a specific intervention, and the literature still lacks studies of dose-response interactions to define rehabilitations gains peak (38). Fourth, the assessor was not blinded to the treatment and, although he could not have access to the patients' previous evaluation results, he may have been led to give higher scores at T1 and T2. To reduce this risk of biases, the patient's referent physician double-checked the clinical evaluation results with the support of the patients' assessments videos; nevertheless, effect sizes might have been inflated, and they should be taken cautiously. Fifth, some hypotheses we made on neuroplasticity to explain the results obtained should be verified by imaging investigations. We made a preliminary study with electroencephalography that seems to confirm our hypotheses (31) but, indeed, further studies are needed. Sixth, our conclusions are based on clinical measures only, which are inherently subjective. The kinematic analysis could help to explain more the mechanisms underlying the recovery following the rehabilitation approach presented in this study. Notably, it could be helpful to measure the movement smoothness, which is a measure of inter-joint coordination and, therefore, an indirect measure of the motor control ability (13, 41). This is particularly important as smoothness was demonstrated to be related to brain activity (42), and its analysis could help to understand what patients exactly learn when measuring improvement in quality of motor performance (43, 44). Finally, the robot used is not commercially available for rehabilitation, and, in case the additive value of the invention would be demonstrated, transfer to clinical practice could be difficult. However, in the last 2 years, our team developed, to continue the study, the control of a new robot (UR0, Universal Robots, Denmark), which is available on the market for human-robot cooperation.

## Conclusion

In this article, we presented a novel rehabilitation intervention based on robot fully assisted functional movements against gravity performed at quasi-physiological velocity. The compliance with the intervention was excellent. Preliminary evidence was provided in chronic stroke for reduced impairment, sustained even at 6-month follow-up. This conclusion is based on the upper-limb FMA. Very preliminary results suggested that reduced impairment translates to improved activity. This conclusion is based on the WMFT and MAL scores. In short, the results are encouraging and lay the foundation for further studies corroborated by imaging and instrumental assessments to confirm the findings, verify the potential additive value of the intervention, and define the optimal dose-response curve. This would be the first step to make attractive for companies the development of a robot enabling this intervention and, therefore, the first step toward clinical use.

## Data Availability Statement

Average groups data are reported in the tables included in the article; further inquiries can be directed to the corresponding author.

## Ethics Statement

The studies involving human participants were reviewed and approved by Comitato Etico Interaziendale delle Province di Lecco, Como e Sondrio. The patients/participants provided their written informed consent to participate in this study.

## Author Contributions

MC participated in the design of the study, data collection, data analyses, and drafted the manuscript. CG and GG participated in the design of the study, enrollment of the patients, and data collection. FM and LM participated in the design and coordination of the study. All authors have read and approved the manuscript.

## Funding

Writing of this study was possible; thanks to funding from the European Union's Horizon 2020 Research and Innovation program *via* an Open Call issued and executed under Project EUROBENCH (grant agreement no. 779963). The study was partially financially supported by the Italian Lombardy Region within the RIPRENDO@home project (D.G.R. no. 3728 - July 11, 2012). All material relating to the study may be disclosed in respect of people's privacy and the World Medical Association Declaration of Helsinki.

## Conflict of Interest

The authors declare that the research was conducted in the absence of any commercial or financial relationships that could be construed as a potential conflict of interest.

## Publisher's Note

All claims expressed in this article are solely those of the authors and do not necessarily represent those of their affiliated organizations, or those of the publisher, the editors and the reviewers. Any product that may be evaluated in this article, or claim that may be made by its manufacturer, is not guaranteed or endorsed by the publisher.
